# Factors Predicting COVID-19 Vaccination Uptake Among Men Who Have Sex With Men in China: An Observational Prospective Cohort Study

**DOI:** 10.3389/fmed.2022.838973

**Published:** 2022-03-11

**Authors:** Kechun Zhang, Paul Shing-fong Chan, Siyu Chen, Yuan Fang, He Cao, Hongbiao Chen, Tian Hu, Yaqi Chen, Xiaofeng Zhou, Zixin Wang

**Affiliations:** ^1^Longhua District Center for Disease Control and Prevention, Shenzhen, China; ^2^Jockey Club (JC) School of Public Health and Primary Care, The Chinese University of Hong Kong, Hong Kong, Hong Kong SAR, China; ^3^Department of Health and Physical Education, The Education University of Hong Kong, Hong Kong, Hong Kong SAR, China

**Keywords:** COVID-19 vaccination, men who have sex with men (MSM), predictors, observational prospective cohort study, China

## Abstract

**Background:**

The coronavirus disease 2019 (COVID-19) pandemic has disproportionately affected sexual minorities including men who have sex with men (MSM). This study investigated factors associated with the uptake of COVID-19 vaccination among MSM in China.

**Methods:**

Inclusion criteria were: (1) born biologically male, (2) had oral or anal sex with men in the past year, (3) aged at least 18 years, and (4) lived in Shenzhen, China. Prospective participants were recruited through outreach in gay bars and saunas, online recruitment, and peer referrals. Participants completed a baseline online survey between August and September 2020 and a follow-up online survey between April and May 2021. Logistic regression models were fitted for data analysis.

**Results:**

Among 420 participants who completed the baseline survey, 303 completed the follow-up survey. Among participants being followed up, 113 (37%) received at least one dose of COVID-19 vaccination during the study period. After adjusting for significant sociodemographic characteristics, five baseline predisposing factors predicted COVID-19 vaccination uptake during the follow-up period, including asking whether their partners had COVID-19 symptoms [adjusted odds ratio (AOR): 1.17, 95% confidence interval (CI): 1.00–1.38], washing hands before and after sex (AOR: 1.23, 95% CI: 1.03–1.46), sanitizing before and after sex (AOR: 1.17, 95% CI: 1.00–1.37), perceived higher risk of COVID-19 transmission through sexual behaviors (AOR: 1.28, 95% CI: 1.04–1.58), and panic about COVID-19 (AOR: 1.48, 95% CI: 1.16–1.89). Regarding enabling factors, receiving testing for sexually transmitted infections (STI) (AOR: 2.19, 95% CI: 1.25–3.85) and other prevention measures for human immunodeficiency virus (HIV)/STI (AOR: 2.61, 95% CI: 1.56–4.37) 6 months prior to the baseline survey were associated with higher uptake of COVID-19 vaccination.

**Conclusion:**

MSM’s uptake rate of COVID-19 vaccination was comparable to that of the general population in Shenzhen, China. This study offered an overview for us to identify tapping points that can encourage COVID-19 vaccination uptake among Chinese MSM.

## Introduction

Globally, coronavirus disease 2019 (COVID-19) imposes a heavy disease burden and an unprecedented challenge to the healthcare system. As immunization is one of the most successful and cost-effective health interventions to prevent infectious diseases, vaccination against COVID-19 is of great importance to prevent and control COVID-19 (1, 2).

China initiated the nationwide COVID-19 vaccination program in December 2020. The country has accelerated the promotion of COVID-19 vaccination for its whole population since late March 2021. In Shenzhen where the study was conducted, people could make an appointment to receive a COVID-19 vaccination. People first downloaded a smartphone application developed by the health bureau. After logging in, they could choose the time and location to receive the vaccine. At the beginning of rollout, only two types of inactivated COVID-19 vaccines were available in China (Sinopharm, and Sinovac CoronaVac). The number of available vaccines increased to six in July 2021, including four inactivated vaccines (Sinopharm, Sinovac CoronaVac, Minhai KCONVAC, and Covidful), one adenovirus vector vaccine (CanSino: Ad5-nCoV), and one subunit vaccine (Zhifei Bio-tech: ZIFIVAX) (3–5). By the end of our study period (May 2021), China provided 661 million doses of COVID-19 vaccines to its 1.3 billion people (6), 41% of people in Shenzhen received a COVID-19 vaccination (7).

The COVID-19 pandemic has disproportionately affected sexual minorities including men who have sex with men (MSM) (8). MSM was one of the vulnerable groups having a high risk of human immunodeficiency virus (HIV) infection and several other health conditions (e.g., cancer, kidney disease, heart disease, hypertension, and stroke) (9, 10). These conditions were associated with an increased risk of developing severe COVID-19 (9, 10). Moreover, MSM experienced higher levels of negative economic impacts (11), poorer mental health (11, 12), and limited access to health services during the pandemic as compared to the general population (11–13). In addition, MSM experienced health disparities associated with stigma and discrimination related to their sexual orientation (14–16), which resulted in medical distrust (17, 18). Previous studies showed that the history of stigma and discrimination was a barrier to taking up human papillomavirus (HPV) vaccination (19), and to using general healthcare services among MSM (18, 20). In China, MSM may experience additional stigma due to cultural factors. Traditional family-oriented values in filial piety, heterosexual marriage, and having children are emphasized in Chinese men’s social roles and duties (21). Additionally, being different from the heterosexual norm is thought of as being abnormal which in the Chinese context implies a problematic form of deviance (22). Studies showed that stigma related to homosexual activities and HIV/sexually transmitted infection (STI) was a barrier against MSM seeking health services in China (23, 24). For instance, in one study conducted in Chengdu, medical distrust was reported by participants that they worried about the stigma and discrimination from healthcare providers and thus avoided seeking health services to avert embarrassment, stigma and discrimination (24). A systematic review reported that bad experiences with healthcare providers (e.g., stigma) contributed to COVID-19 vaccination hesitancy among sexual minorities (25). In an attempt to address health disparity issues for COVID-19 vaccination, the World Health Organization (WHO) have placed great emphasis on equal access and distribution of COVID-19 vaccines (26), and the rights and interests of equitable access to COVID-19 vaccines of sexual minorities have been recognized by the United States National Academies of Sciences, Engineering, and Medicine (27).

Two studies looked at MSM’s willingness to receive a COVID-19 vaccination in the United States, and the prevalence of willingness was 63 and 78%, respectively (13, 28). One study conducted in Taiwan, China showed that sexual minority individuals had higher levels of intention to receive a COVID-19 vaccination as compared to heterosexual individuals (29). Being HIV positive, using preventive measures during sexual behaviors (e.g., having reduced sex partners) and experiences related to COVID-19 (e.g., having friends and sex partners infected with COVID-19) were associated with willingness to receive a COVID-19 vaccination among MSM in the United States (28).

The Predisposing, Reinforcing and Enabling Constructs in Educational/Ecological Diagnosis and Evaluation (PRECEDE) model provided a theoretical framework to identify predictors of COVID-19 vaccination uptake in this study (30). It is one of the oldest and most enduring models used in health promotion which was developed and introduced in the 1970s (26). It was used to improve influenza vaccination (31), breast self-examination (32), weight management (33), quality of life (34), and oral health (35) in previous studies. This model posits that health behaviors (e.g., COVID-19 vaccination uptake) are influenced by predisposing factors (characteristics that lead to or motivate behavior, including perceptions and psychosocial variables), enabling factors (characteristics that facilitate or are needed to perform the behavior), and reinforcing factors (rewards and punishments). Regarding predisposing factors, both systematic reviews on individuals’ willingness and actual uptake of COVID-19 vaccination among the general population suggested that higher levels of panic and perceived risk of COVID-19 were facilitators (36, 37). Many MSM remained sexually active during COVID-19 (38, 39), including those in mainland China (40). MSM concerned about the risk of COVID-19 transmission through sexual or other intimate behaviors (41, 42). Such concerns may motivate them to receive a COVID-19 vaccination. Moreover, MSM experienced elevated rates of mental health problems and stress as compared to their heterosexual counterparts across geographic areas (43, 44). The COVID-19 pandemic aggravated mental health problems and stress among both the general population and MSM (45–47). Poorer mental health status was a barrier to receiving health services among MSM (48), and was a barrier to receiving a COVID-19 vaccination in other populations (49). However, the association between mental health and COVID-19 vaccination uptake has not been studied among MSM. Regarding enabling factors, we considered access to other health services and COVID-19-related experiences (e.g., COVID-19 infection, centralized/home quarantine).

Nonetheless, to our knowledge, there is no study conducted to investigate factors associated with the uptake of COVID-19 vaccination among MSM. To address the knowledge gaps, we conducted a prospective observational cohort study among a sample of MSM in Shenzhen, China between August 2020 and May 2021. Participants completed online surveys at baseline and 9 months afterward. We aimed to investigate baseline factors predicting COVID-19 vaccination uptake during the follow-up period among MSM, including sociodemographic characteristics, predisposing factors, and enabling factors. Regarding predisposing factors at baseline, we hypothesized that individuals who had sexual risk behaviors, a higher frequency of using COVID-19 preventive measures during sexual behaviors, a higher level of COVID-19 risk perception, a higher level of panic, and a lower level of mental health problems would be more likely to receive COVID-19 vaccination. For enabling factors, we hypothesized that individuals who utilized HIV-related services in the past 6 months and had more experiences related COVID-19 would be more likely to receive COVID-19 vaccination.

## Materials and Methods

### Study Design and Participants

A prospective observational cohort study was conducted among MSM in Shenzhen, China. Participants completed a baseline online self-administered survey between August and September 2020, and a follow-up survey between April and May 2021.

Participants were: (1) born biologically male, (2) aged at least 18 years, (3) Chinese speaking, (4) living in Shenzhen, (5) having oral or anal sex with men in the past year, and (6) willing to leave contact information and complete the follow-up survey.

### Recruitment Process and Data Collection

**FIGURE 1:**
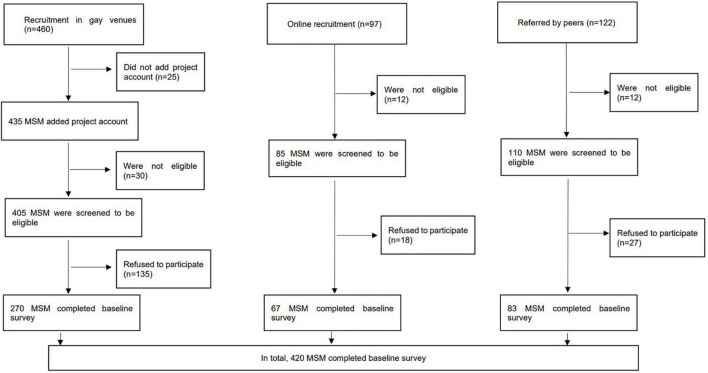
Flowchart of the study.

We recruited participants from multiple channels. Prospective participants were approached by experienced staff in places that were frequently visited by MSM, such as gay bars and gay saunas, at different time slots. In addition, the research team conducted web-based outreaches by periodically posting information about the study on two commonly used social media platforms in China, i.e., Weibo and WeChat. Recruitment was supplemented by peer referrals. Prospective participants were briefed on study details on-site or *via* telephone/live chat applications. The fieldworkers approached 460 prospective participants in gay venues, 435 added the project official WeChat account, 405 were screened eligible through WeChat, 135 refused to participate and 270 completed the baseline survey. Regarding online recruitment, 97 prospective participants contacted the fieldworkers, 85 were screened to be eligible, 18 refused to participate, and 67 completed the baseline survey. Among 122 prospective participants referred by peers, 110 were screened to be eligible, 27 refused to participate and 83 completed the baseline survey. In sum, 420 participants completed the baseline survey with an overall response rate of 70%. A total of 303 participants completed the follow-up survey, the drop-out rate was 27.9%. A flowchart of recruitment was shown in Figure 1. Participants were guaranteed anonymity during the study and had the right to discontinue participation in the study at any time. Their refusal or withdrawal from the study would not affect their access to any future services. Participants signed an electronic consent form sent by WeChat.

Baseline and follow-up surveys were completed through mobile devices, and survey data were collected through the online survey platform. The online surveys were developed by using Questionnaire Star, a commonly used online survey platform in China. A Quick Response (QR) code to link to the survey was generated, and participants could scan the QR code to complete the survey. The online survey platform could restrict the use of the Internet Protocol (IP) addresses to ensure that all participants completed the survey only once and avoid repeated responses. The baseline survey consisted of 105 items, which took about 20 min to complete. The follow-up survey had 60 items, which took about 10 min to complete. Prior to submission of the survey, the Questionnaire Star would check its completeness. An e-coupon of 20 Chinese Yuan (approximately US$3.0) was sent to participants upon completion of each survey as compensation for their time.

### Measures

#### Design of the Survey

A panel consisting of three staff from Longhua District Center for Disease Control and Prevention (CDC), two public health researchers, a health psychologist, and two MSM volunteers was formed to develop the surveys used in this study. The surveys were pilot tested among 10 MSM to assess clarity and readability. All MSM in the pilot testing agreed that the questions were easy to understand and the length was appropriate. These 10 MSM did not participate in the study. Based on their comments, the panel revised and finalized the surveys.

#### COVID-19 Vaccination Uptake

At Month 9, participants were asked whether they had taken up any COVID-19 vaccine during the follow-up period. Some supplementary information was collected from the vaccinated participants, including the number of doses and types of COVID-19 vaccines received, presence of side effects, and severity of these side effects.

#### Sociodemographic Variables

Information on socio-demographics was collected, including age, current relationship status, highest education level attained, current employment status, monthly personal income, sexual orientation, and HIV sero-status.

#### Predisposing Factors Measured at Baseline

Participants were asked to report whether they had condomless anal intercourse (CAI) with male regular sex partners (RP) and male non-regular sex partners (NRP), male sex workers, and sexualized drug use (SDU) in 6 months prior to the baseline survey. RP was defined as their lovers or stable boyfriends. NRP was defined as casual sex partners, while male sex workers were defined as males providing sexual services in return for payment. SDU was defined as the use of any of the following psychoactive substances [i.e., ketamine, methamphetamine, cocaine, cannabis, ecstasy, Dormicum/Halcion/Erimin 5/non-prescription hypnotic drugs, heroin, codeine from cough suppressants, amyl nitrates (poppers), γ-hydroxybutyrate (G water), and Foxy] before or during sexual intercourse (50, 51).

Participants reported the use of the following preventive measures during sexual behaviors 6 months prior to the baseline survey, including: (i) only having sex with my partner who has had sex with me before, (ii) avoiding group sex, (iii) only having sex at home, (iv) only having sex in other places rather than at home, (v) asking your partner if they have COVID-19 symptoms, (vi) avoiding kissing during sex, (vii) washing hands before and after sex, and (viii) sanitizing before and after sex. Response categories for these items were: 1 = never, 2 = seldom, 3 = sometimes, 4 = often, 5 = always.

Perception variables included perceived risk of COVID-19 transmission through sex and other intimate behaviors, the infectiousness of asymptomatic patients, concern that male RP and NRP would transmit COVID-19, and concern about COVID-19 infection when taking up HIV testing. The response categories for these variables were: 1 = strongly disagree, 2 = disagree, 3 = neutral, 4 = agree, and 5 = strongly agree.

Participants were asked about level of panic about COVID-19 and the response categories were 1 = absolutely not, 2 = relatively low, 3 = neutral, 4 = high, and 5 = very high. In addition, the validated Chinese version of the 10-item Centre for Epidemiological Studies Depression Scale (CESD-10) was used to measure participants’ depressive symptoms. The Cronbach’s alpha of the CESD-10 was 0.86 in this study.

#### Enabling Factors Measured at Baseline

Service utilization in the past 6 months was asked, including whether they took testing for HIV and STI, and whether they used other prevention services [e.g., condom distribution, peer education, HIV/acquired immune deficiency syndrome (AIDS) promotion leaflets, and lectures]. Participants were asked whether any of their friends/family members were infected with COVID-19, whether they were infected with COVID-19, and whether they had centralized/home quarantine.

### Sample Size Calculation

The target sample size was 300. Given a statistical power of 0.80 and an alpha value of 0.05 and assuming the prevalence of COVID-19 vaccination uptake in the reference group (without a facilitating condition) to be 10–40%, the sample size could detect a smallest odds ratios of 1.92 between people with and without the facilitating conditions (PASS 11.0, NCSS LLC). Assuming the drop-out rate was 25–30% at follow-up, it was necessary to recruit 400–430 participants at the baseline.

### Ethics Statement

Ethics approval was obtained from the Longhua District Center for Disease Control and Prevention (CDC) (reference: 2021009).

### Statistical Analysis

The difference in baseline characteristics between those who were followed up at Month 9 and those who were lost to follow-up were compared using the chi-square test (X^2^) for categorical variables and independent-sample *t*-test for continuous variables. Using self-reported uptake of at least one dose of COVID-19 vaccination during the follow-up period as the dependent variable, and sociodemographic characteristics as independent variables, crude odds ratio (OR) predicting the dependent variable were obtained using logistic regression models. After adjusting for those sociodemographic characteristics with *p* < 0.05 in the univariate analysis, the associations between the independent variables of interest (e.g., predisposing and enabling factors) and the dependent variable were then obtained by adjusted odds ratio (AOR), and respective 95% confidence interval (CI) were derived from the analyses. Each AOR was obtained by fitting a single logistic regression model, which involved one of the independent variables and the significant sociodemographic variables. SPSS version 26.0 (Chicago, IL, United States) was used for data analysis and *p*-values < 0.05 were considered as statistically significant.

## Results

### Baseline Characteristics

At baseline, over half of the participants were aged 18–30 years (76.7%), currently single (80.2%), had attained college education or above (60.5%), full-time employed (70.7%), had monthly income of less than RMB10000 (US$1537.9) (68.4%), identified themselves as homosexual or bisexual (91.9%), and were HIV negative (76.9%). There were no transgender women in our sample. The prevalence of CAI with RP, NRP, and sex workers was 24.5, 12.9, and 2.6%, respectively, and 11.9% reported sexualized drug use. During sexual behaviors, 11.7–60.5% of participants often/always practiced various COVID-19 preventive measures. For perceived risk of COVID-19 transmission through different channels, 38.8–79.3% agreed/strongly agreed that the risk was high. For psychological variables, the mean score was 3.1 [standard deviation (SD): 1.1] and 21.9 (SD: 6.8) for level of panic about COVID-19 and CESD-10, respectively. Regarding service utilization, 59.5, 24.5, and 36.2% used HIV testing, STI testing, and other HIV/STI prevention services in the past 6 months, respectively. For experiences related to COVID-19, 1.2, 1.7, and 15.7% reported having friends/family members infected with COVID-19, history of COVID-19 infection, and centralized/home quarantine, respectively (Table 1).

**TABLE 1:** Baseline characteristics of participants.

	All participants (*n* = 420)	Follow-up at Month 9 (*n* = 303)	Loss to follow-up (*n* = 117)	
		
	*n* (%)	*n* (%)	*n* (%)	*P* values
**Sociodemographic**				
**Age group (years)**				
18–24	133 (31.7)	88 (29.0)	45 (38.5)	0.11
25–30	189 (45.0)	143 (47.2)	46 (39.3)	
31–40	75 (17.9)	58 (19.1)	17 (14.5)	
>40	23 (5.5)	14 (4.6)	9 (7.7)	
**Current relationship status**				
Currently single	337 (80.2)	239 (78.9)	98 (83.8)	0.50
Married or cohabited with a man	68 (16.2)	53 (17.5)	15 (12.8)	
Married or cohabited with a woman	15 (3.6)	11 (3.6)	4 (3.4)	
**Highest education level attained**				
Junior high or below	56 (13.3)	38 (12.5)	18 (15.4)	0.18
Senior high or equivalent	94 (22.4)	61 (20.1)	33 (28.2)	
College or above	254 (60.5)	193 (63.7)	61 (52.1)	
Others	16 (3.8)	11 (3.6)	5 (4.3)	
**Current employment status**				
Full-time	297 (70.7)	221 (72.9)	76 (65.0)	0.11
Part-time/unemployed/retired/students/others	123 (29.3)	82 (27.1)	41 (35.0)	
**Monthly personal income, Chinese Yuan (US dollar)**				
Below 3000 (461.4)	33 (7.9)	22 (7.3)	11 (9.4)	0.21
3000–4999 (461.4–768.9)	93 (22.1)	63 (20.8)	30 (25.6)	
5000–6999 (769.1–1076.5)	88 (21.0)	58 (19.1)	30 (25.6)	
7000–9999 (1076.7–1537.9)	73 (17.4)	59 (19.5)	14 (12.0)	
10000 or above (1538.1)	76 (18.1)	61 (20.1)	15 (12.8)	
No fixed income	38 (9.0)	27 (8.9)	11 (9.4)	
Refused to disclose	19 (4.5)	13 (4.3)	6 (5.1)	
**Sexual orientation**				
Homosexual	302 (71.9)	238 (78.5)	64 (54.7)	**<0.001**
Bisexual	84 (20.0)	52 (17.2)	32 (27.4)	
Heterosexual	14 (3.3)	3 (1.0)	11 (9.4)	
Uncertain	20 (4.8)	10 (3.3)	10 (8.5)	
**HIV sero-status**				
Negative	323 (76.9)	246 (81.2)	77 (65.8)	**0.005**
Positive	8 (1.9)	6 (2.0)	2 (1.7)	
Refused to disclose	27 (6.4)	17 (5.6)	10 (8.5)	
Had not tested for HIV	62 (14.8)	34 (11.2)	28 (23.9)	
**Predisposing factors**				
**Sexual behaviors in the past 6 months, *n* (%) Yes**				
Condomless anal intercourse with regular male sex partners	103 (24.5)	77 (25.4)	26 (22.2)	0.50
Condomless anal intercourse with non-regular male sex partners	54 (12.9)	41 (13.5)	13 (11.1)	0.51
Condomless anal intercourse with male sex workers	11 (2.6)	6 (2.0)	5 (4.3)	0.19
Sexualized drug use	50 (11.9)	38 (12.5)	12 (10.3)	0.52
**COVID-19 preventive measures during sexual behaviors, *n* (%) often/always**				
Only having sex with my partner who has had sex with me before	141 (33.6)	113 (37.3)	28 (23.9)	**0.009**
Avoiding group sex	172 (41.0)	137 (45.2)	35 (29.9)	**0.004**
Only having sex at home	175 (41.7)	138 (45.5)	37 (31.6)	**0.009**
Only having sex in other places rather than at home	49 (11.7)	36 (11.9)	13 (11.1)	0.83
Asking your partner whether they have COVID-19 symptoms	112 (26.7)	91 (30.0)	21 (17.9)	**0.01**
Avoiding kissing during sex	106 (25.2)	87 (28.7)	19 (16.2)	**0.008**
Washing hands before and after sex	254 (60.5)	198 (65.3)	56 (47.9)	**0.001**
Sanitizing before and after sex	174 (41.4)	139 (45.9)	35 (29.9)	**0.003**
**Perceptions related to COVID-19, *n* (%) agree/strongly agree**				
The risk of COVID-19 transmission through sexual behavior is high	241 (57.4)	176 (58.1)	65 (55.6)	0.64
The risk of COVID-19 transmission through other intimate behaviors (e.g., kissing, touching) is high	333 (79.3)	245 (80.9)	88 (75.2)	0.20
Asymptomatic patients have a high risk of transmitting COVID-19	232 (55.2)	170 (56.1)	62 (53.0)	0.57
Your regular male sex partner would transmit COVID-19 to you	190 (45.2)	142 (46.9)	48 (41.0)	0.28
Your non-regular male sex partner would transmit COVID-19 to you	236 (56.2)	178 (58.7)	58 (49.6)	0.09
You concern about COVID-19 infection when taking up HIV testing	163 (38.8)	119 (39.3)	44 (37.6)	0.75
**Psychological variables, mean (SD)**				
Level of panic about COVID-19	3.1 (1.1)	3.1 (1.0)	3.0 (1.2)	0.33
CESD-10	21.9 (6.8)	22.0 (6.6)	21.6 (7.3)	0.59
**Enabling factors**				
**Service utilization in the past 6 months, *n* (%) Yes**				
Any type of HIV testing	250 (59.5)	190 (62.7)	60 (51.3)	**0.03**
Testing for STI	103 (24.5)	80 (26.4)	23 (19.7)	0.15
Other HIV/STI prevention services^1^	152 (36.2)	115 (38.0)	37 (31.6)	0.23
**Experiences related to COVID-19, *n* (%) Yes**				
Friends/family members infected with COVID-19	5 (1.2)	2 (0.7)	3 (2.6)	0.14
History of COVID-19 infection	7 (1.7)	4 (1.3)	3 (2.6)	0.40
Centralized/home quarantine	66 (15.7)	57 (18.8)	9 (7.7)	**0.005**

*AIDS, acquired immune deficiency syndrome; HIV, human immunodeficiency virus; SD, standard deviation; STI, sexually transmitted infection. ^1^Other HIV/STI prevention services: condom distribution, peer education, HIV/AIDS promotion leaflets and lectures, and HIV/AIDS prevention knowledge via the Internet or social media. P values were obtained using the chi-square test (X^2^) for categorical variables or independent-sample t-tests for continuous variables. The bold values are statistically significant with P < 0.05.*

As compared to those who were lost to follow-up (*n* = 117), participants who were followed up (*n* = 303) were more likely to identify themselves as homosexual (*p* < 0.001), be HIV negative (*p* = 0.005), only have sex with the partners who had sex with them before (*p* = 0.009), avoid group sex (*p* = 0.004), only have sex at home (*p* = 0.009), ask their partners if they had COVID-19 symptoms (*p* = 0.01), avoid kissing during sex (*p* = 0.008), wash hands before and after sex (*p* = 0.001), and sanitize before and after sex (*p* = 0.003), be tested for HIV in the past 6 months (*p* = 0.03), and have centralized/home quarantine (*p* = 0.005) (Table 1).

### COVID-19 Vaccination Uptake

Among the 303 participants who completed the follow-up survey, 37.3% (*n* = 113) received at least one dose of the COVID-19 vaccination; 77 and 36 participants received one dose and two doses, respectively. Among vaccinated participants, 23.0% received Sinopharm, 44.2% received Sinovac CoronaVac, 4.4% received CanSino: Ad5-nCoV, and 28.3% were not sure about the type of vaccine. Regarding side effects of COVID-19 vaccination, 26.5% had pain at the injection site, 1.8% had redness/itching/induration/rash at the injection site, 13.3% had fatigue/headache/dizziness/drowsiness, and 6.2% had muscle/joint pain. Itching at the non-injection site (1.8%), fever (0.9%), nausea/vomiting/diarrhea (2.7%) were rare. Most of these side-effects were very mild/mild (90.2%).

### Factors Predicting Uptake of at Least One Dose of COVID-19 Vaccination During the Follow-Up Period

In univariate analysis, participants who were above 40 years old and married/cohabited with a man were more likely to receive COVID-19 vaccination. Participants who identified themselves as bisexual were less likely to receive COVID-19 vaccination (Table 2).

**TABLE 2:** Associations between sociodemographic characteristics and uptake of COVID-19 vaccination (at least one dose) during the follow-up period (among participants who completed both surveys, *n* = 303).

	OR (95%CI)	*P* values
**Sociodemographic**		
**Age group (years)**		
18–24	1.00	
25–30	0.96 (0.55–1.67)	0.88
31–40	1.21 (0.61–2.40)	0.59
>40	4.60 (1.33–15.87)	**0.02**
**Current relationship status**		
Currently single	1.00	
Married or cohabited with a man	2.14 (1.18–3.91)	**0.01**
Married or cohabited with a woman	0.72 (0.19–2.78)	0.63
**Highest education level attained**		
Junior high or below	1.00	
Senior high or equivalent	1.41 (0.60–3.31)	0.44
College or above	1.38 (0.66–2.89)	0.40
Others	0.48 (0.09–2.58)	0.39
**Current employment status**		
Full-time	1.00	
Part-time/unemployed/retired/students/others	1.11 (0.66–1.86)	0.70
**Monthly personal income, Chinese Yuan (US dollar)**		
Below 3000 (461.4)	1.00	
3000–4999 (461.4–768.9)	0.69 (0.26–1.85)	0.46
5000–6999 (769.1–1076.5)	0.68 (0.25–1.84)	0.45
7000–9999 (1076.7–1537.9)	0.88 (0.33–2.36)	0.80
10000 or above (1538.1)	0.68 (0.25–1.82)	0.44
Refused to disclose	0.60 (0.19–1.91)	0.39
No fixed income	0.36 (0.08–1.68)	0.19
**Sexual orientation**		
Homosexual	1.00	
Bisexual	0.39 (0.19–0.80)	**0.01**
Heterosexual	0.73 (0.07–8.13)	0.80
Uncertain	0.97 (0.27–3.53)	0.96
**HIV sero-status**		
Negative	1.00	
Positive	0.31 (0.04–2.72)	0.29
Refused to disclose	0.65 (0.22–1.91)	0.43
Had not tested for HIV	0.75 (0.35–1.60)	0.45

*CI, confidence interval; HIV, human immunodeficiency virus; OR, crude odds ratio. The bold values are statistically significant with P < 0.05.*

After adjusting for these significant sociodemographic characteristics, five predisposing factors predicted COVID-19 vaccination uptake during the follow-up period. These factors were asking whether their partners had COVID-19 symptoms (AOR: 1.17, 95% CI: 1.00–1.38, *p* = 0.046), washing hands before and after sex (AOR: 1.23, 95% CI: 1.03–1.46, *p* = 0.02), sanitizing before and after sex (AOR: 1.17, 95% CI: 1.00–1.37, *p* = 0.048), perceived higher risk of COVID-19 transmission through sexual behaviors (AOR: 1.28, 95% CI: 1.04–1.58, *p* = 0.02), and panic about COVID-19 (AOR: 1.48, 95% CI: 1.16–1.89, *p* = 0.002). Regarding enabling factors, receiving STI testing (AOR: 2.19, 95% CI: 1.25–3.85, *p* = 0.006) and other HIV/STI prevention services (AOR: 2.61, 95% CI: 1.56–4.37, *p* < 0.001) were associated with higher uptake of COVID-19 vaccination (Table 3).

**TABLE 3:** Factors associated with uptake of COVID-19 vaccination (at least one dose) during the follow-up period (among participants who completed both surveys, *n* = 303).

	OR (95%CI)	*P* values	AOR (95%CI)	*P* values
**Predisposing factors**				
**Sexual behaviors in the past 6 months**				
**Condomless anal intercourse with regular male sex partners**				
No	1.00		1.00	
Yes	1.70 (1.01–2.87)	**0.048**	1.56 (0.89–2.73)	0.12
**Condomless anal intercourse with non-regular male sex partners**				
No	1.00		1.00	
Yes	0.85 (0.43–1.71)	0.65	0.74 (0.35–1.55)	0.42
**Condomless anal intercourse with male sex workers**				
No	1.00		1.00	
Yes	0.33 (0.04–2.86)	0.32	0.42 (0.05–3.78)	0.44
**Sexualized drug use**				
No	1.00		1.00	
Yes	1.11 (0.55–2.23)	0.77	1.13 (0.54–2.35)	0.74
**COVID-19 preventive measures during sexual behaviors**				
Only having sex with my partner who has had sex with me before	1.08 (0.93–1.26)	0.31	1.08 (0.92–1.27)	0.35
Avoiding group sex	0.99 (0.87–1.12)	0.83	0.99 (0.87–1.13)	0.91
Only having sex at home	1.06 (0.90–1.24)	0.50	1.05 (0.89–1.24)	0.54
Only having sex in other places rather than at home	1.08 (0.89–1.30)	0.43	1.15 (0.94–1.40)	0.18
Asking your partner if they have COVID-19 symptoms	1.13 (0.98–1.31)	0.10	1.17 (1.00–1.38)	**0.046**
Avoiding kissing during sex	0.99 (0.84–1.16)	0.86	1.02 (0.86–1.20)	0.87
Washing hands before and after sex	1.20 (1.02–1.42)	**0.03**	1.23 (1.03–1.46)	**0.02**
Sanitizing before and after sex	1.13 (0.98–1.31)	0.09	1.17 (1.00–1.37)	**0.048**
**Perceptions related to COVID-19**				
The risk of COVID-19 transmission through sexual behavior is high	1.26 (1.04–1.54)	**0.02**	1.28 (1.04–1.58)	**0.02**
The risk of COVID-19 transmission through other intimate behaviors (e.g., kissing, touching) is high	1.13 (0.89–1.43)	0.31	1.50 (0.79–2.86)	0.21
Asymptomatic patients have a high risk of transmitting COVID-19	1.20 (0.95–1.51)	0.13	1.26 (0.77–2.06)	0.37
Your regular male sex partner would transmit COVID-19 to you	1.04 (0.86–1.26)	0.67	1.00 (0.61–1.63)	0.99
Your non-regular male sex partner would transmit COVID-19 to you	1.03 (0.84–1.27)	0.75	0.87 (0.53–1.44)	0.59
You concern about COVID-19 infection when taking up HIV testing	1.05 (0.87–1.26)	0.65	0.91 (0.55–1.50)	0.71
**Psychological variables, mean (SD)**				
Level of panic about COVID-19	1.42 (1.12–1.79)	**0.003**	1.48 (1.16–1.89)	**0.002**
CESD-10	0.96 (0.93–0.99)	**0.04**	0.97 (0.93–1.01)	0.13
**Enabling factors**				
**Service utilization in the past 6 months**				
**Any type of HIV testing**				
No	1.00		1.00	
Yes	1.14 (0.70–1.85)	0.60	1.13 (0.68–1.88)	0.64
**Testing for STI**				
No	1.00		1.00	
Yes	1.79 (1.06–3.00)	**0.03**	2.19 (1.25–3.85)	**0.006**
**Other HIV/STI prevention services^1^**				
No	1.00		1.00	
Yes	2.46 (1.52–3.99)	**<0.001**	2.61 (1.56–4.37)	**<0.001**
**Experiences related to COVID-19**				
**Friends/family members infected with COVID-19**				
No	1.00		1.00	
Yes	1.69 (0.11–27.25)	0.71	1.92 (0.12–31.62)	0.65
**History of COVID-19 infection**				
No	1.00		1.00	
Yes	5.16 (0.53–50.16)	0.16	12.85 (0.88–186.89)	0.06
**Centralized/home quarantine**				
No	1.00		1.00	
Yes	1.28 (0.71–2.31)	0.41	1.18 (0.64–2.18)	0.59

*AOR, adjusted odds ratio, odds ratio obtained by adjusting for significant sociodemographic variables; CESD-10, 10-item Centre for Epidemiological Studies Depression Scale; CI, confidence interval; HIV, human immunodeficiency virus; OR, crude odds ratio; STI, sexually transmitted infection. The bold values are statistically significant with P < 0.05. ^1^Condom distribution, peer education, HIV/AIDS promotion leaflets and lectures, and HIV/AIDS prevention knowledge via the Internet or social media.*

## Discussion

To our knowledge, this is one of the first studies investigating COVID-19 vaccination uptake among MSM. The findings represented the latest estimate of vaccination coverage in this group. This study used the prospective longitudinal study design to investigate the association between baseline factors and COVID-19 vaccination uptake during the follow-up period. Causal relationships could be established. The findings suggested that predisposing and enabling factors measured at baseline significantly predicted COVID-19 vaccination uptake, which expanded the application of the PRECEDE model.

About 40% of the sampled MSM received COVID-19 vaccination during the follow-up period. Such uptake rate was comparable to that of the general population in Shenzhen (41%) in May 2021 (7). Those aged over 40 years reported the highest COVID-19 vaccination uptake, this finding was similar to that reported in the general population in China and other countries (52, 53). Older age is a significant predictor of COVID-19 mortality and severe complications (54, 55). It is possible that older MSM are more likely to consider COVID-19 as a serious health threat, and hence are more motivated to receive a COVID-19 vaccination. More attention should be given to young MSM in future health programs. Similar to previous studies, MSM who were married or cohabited with a man reported higher uptake (56). Protecting one’s male partners might be a motivation to receive a COVID-19 vaccination among MSM. In addition, more attention should be given to bisexual men as they reported lower COVID-19 vaccination uptake.

Regarding predisposing factors, the majority of our sampled MSM remained sexually active during the pandemic which was in line with other studies (38, 40). As a result, about 60% of them perceived a high risk of COVID-19 transmission through sexual behaviors. Future programs should emphasize the risk of COVID-19 transmission through sexual behaviors, as such perception was associated with higher uptake of COVID-19 vaccination among MSM. MSM taking COVID-19 preventive measures (checking partners’ COVID-19 symptoms, washing hands and sanitizing before/after sex) were more likely to take up COVID-19 vaccination. It is possible that they have higher self-efficacy and motivation to protect themselves against COVID-19, and they might consider COVID-19 vaccination as an effective means of prevention. In addition, a higher level of panic about COVID-19 was associated with a higher uptake echoes the results obtained in the general population (57, 58). However, it is not ethical to enhance panic as it would lead to mental health problems. Future programs should emphasize that taking up COVID-19 vaccination is an effective way to reduce panic caused by COVID-19.

Utilization of STI testing, and other HIV/STI prevention services were enabling factors associated with higher COVID-19 vaccination uptake among MSM. It is possible that MSM who accessed these services had a lower level of medical distrust (17, 18). Previous studies observed a similar association between HIV/STI prevention service utilization and HPV vaccination uptake among MSM (59). Therefore, community-based organization (CBO) in China has a good position of promoting COVID-19 vaccination among MSM. First, most Chinese MSM usually received HIV/STI prevention services provided by CBO (60, 61). Second, suggestions from peer CBO workers can serve as a strong cue to action to take up COVID-19 vaccination (62). MSM in China are more likely to value peers’ opinions.

Our study had several limitations. First, some important factors, such as stigma and discrimination related to their sexual identity and reinforcing factors under the PRECEDE model (e.g., rewards and punishment) were not measured by this study. Second, attrition bias existed. As compared to those who were followed up, dropouts had lower utilization of HIV testing, were less likely to check partners’ COVID-19 symptoms, and washing hands and sanitizing hands before/after sex. It was likely that the COVID-19 vaccination uptake was overestimated in this study. Third, we were not able to obtain the characteristics of MSM who refused to join this study. Participants and refusals might have different characteristics. Selection bias existed. Moreover, this study was conducted in one Chinese city and the participants could not represent all MSM in China. COVID-19 vaccination was first scaled up in first-line Chinese cities, such as Shenzhen. It was possible that MSM in other Chinese cities would have lower COVID-19 vaccination uptake as compared to their counterparts in Shenzhen. Furthermore, there are more organizations providing HIV prevention services for MSM in Shenzhen, as compared to other cities with general economy or lower. Therefore, generalization of the results to other parts of China should be made with caution. Lastly, some measurements were self-constructed for this study. They were not validated externally.

## Conclusion

About 40% of MSM received COVID-19 vaccination during the follow-up period. Using the PRECEDE model, this study offered an overview for us to identify tapping points that can encourage COVID-19 vaccination uptake among Chinese MSM. Future health promotion could be delivered by CBO providing HIV/STI prevention service, and addressing their concern about the risk of COVID-19 transmission during sexual behaviors.

## Data Availability Statement

The raw data supporting the conclusions of this article will be made available by the authors, without undue reservation.

## Ethics Statement

The studies involving human participants were reviewed and approved by Longhua District Center for Disease Control and Prevention (CDC) (reference: 2021009). The patients/participants provided their online informed consent to participate in this study.

## Author Contributions

KZ and ZW: conceptualization, methodology, and supervision. KZ, HCa, HCh, TH, YC, and XZ: data curation and project administration. ZW, PC, and SC: formal analysis. ZW, YF, PC, and SC: writing—original draft preparation and writing—review and editing. All authors have read and agreed to the published version of the manuscript.

## Conflict of Interest

The authors declare that the research was conducted in the absence of any commercial or financial relationships that could be construed as a potential conflict of interest.

## Publisher’s Note

All claims expressed in this article are solely those of the authors and do not necessarily represent those of their affiliated organizations, or those of the publisher, the editors and the reviewers. Any product that may be evaluated in this article, or claim that may be made by its manufacturer, is not guaranteed or endorsed by the publisher.
